# Bioactive Compounds from Marine Organisms: Potential for Bone Growth and Healing

**DOI:** 10.3390/md16090340

**Published:** 2018-09-18

**Authors:** Matthew A. Carson, Susan A. Clarke

**Affiliations:** School of Nursing and Midwifery, Queen’s University Belfast, Belfast BT9 7BL, UK; s.a.clarke@qub.ac.uk

**Keywords:** marine, bioactive, compound, extract, bone, bone growth, bone healing, osteoporosis, algae, nacre

## Abstract

Marine organisms represent a highly diverse reserve of bioactives which could aid in the treatment of a wide range of diseases, including various musculoskeletal conditions. Osteoporosis in particular would benefit from a novel and effective marine-based treatment, due to its large disease burden and the inefficiencies of current treatment options. Osteogenic bioactives have been isolated from many marine organisms, including nacre powder derived from molluscan shells and fucoidan—the sulphated polysaccharide commonly sourced from brown macroalgae. Such extracts and compounds are known to have a range of osteogenic effects, including stimulation of osteoblast activity and mineralisation, as well as suppression of osteoclast resorption. This review describes currently known soluble osteogenic extracts and compounds from marine invertebrates and algae, and assesses their preclinical potential.

## 1. Introduction

Globally, an osteoporosis-related fracture occurs once every 3 seconds [1], whilst in Europe the disease burden exceeds that of common neoplastic disorders, excluding lung cancer [1]. Osteoporosis is linked to abnormal functioning of the basic multicellular unit (BMU); with decreased bone formation by osteoblasts and increased resorption by osteoclasts, meaning bone is weaker and more susceptible to fracture damage [2,3,4]. Fractures tend to affect people in their fourth or fifth decade of life, particularly postmenopausal women, are often painful [5] and slow to heal, and present a large healthcare burden [6]. Current treatment options are limited, having issues with their efficacy and long-term use. For example, the antiresorptive drugs, bisphosphonates, are effective at reducing fracture risk [7,8], but only promote small increases in bone density (<2%) [9] and have a number of rare but severe side effects [10]. Anabolic agents, which increase bone growth, are even more limited—with teriparatide the only clinically available treatment option. However, teriparatide requires daily administration and is very costly, with positive effects quickly lost after ceasing treatment [10,11]. As such, there is a need for new and efficacious treatment options, able to stimulate the recovery of bone density and structure. Additionally, there are a number of other conditions, such as complex fracture and malunion, that would also benefit from this [12].

Treatment for bone defects is highly variable, though in general bone regenerative medicine has a triad of key features required for healing: a cellular component, a scaffold for tissue growth, and the inclusion of bioactive/growth factors [13]. Excluding the cellular component, marine organisms are a potential source of scaffold material and novel osteogenic (promoting new osteoblast bone formation) bioactives. Scaffolds and other marine structural components used in tissue engineering are beyond the scope of this review, though show significant promise and have been included in other comprehensive reviews [14,15]. Instead, this work will detail bioactives derived from a diverse range of marine taxa. These organisms produce bioactives through specialisation and adaptation, driven by the challenge of living in environments such as the littoral zone [16,17]; with its extremes of variables such as light, nutrient and temperature levels. Typically, marine environments are less well studied than their terrestrial counterparts [16,18], likely due to the time and cost implications of exploratory work [19]. However, there are examples of marine-derived osteogenic compounds/molecules that show excellent clinical potential [20], and work in this area is continually developing—justifying an up-to-date review of this field.

**Figure 1:**
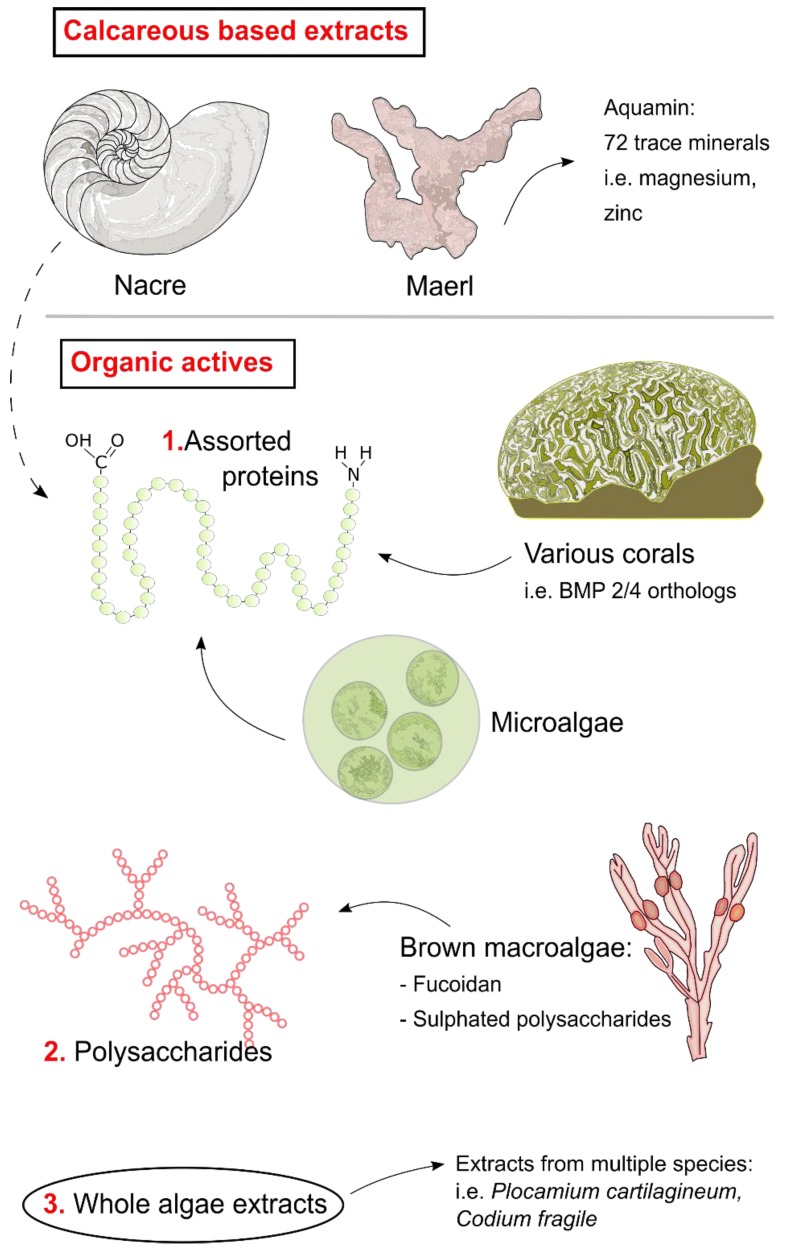
Summary figure highlighting some of the most promising marine extracts and bioactives with osteogenic activity. Two groups are included—calcareous based extracts (nacre and maerl) and organic extracts, which contains three main groups: assorted proteins, polysaccharides, and whole algae extracts.

The aim of this review is to summarise the current marine organism-derived bioactives with osteogenic potential. It will include discussion of invertebrate and alga-derived material (which show particular promise), but will exclude bioactives from marine vertebrates such as fish (see Table 1 and Figure 1 for summary of extracts included). Challenges to undertaking this work and sourcing marine bioactives will also be discussed, as will future recommendations for the field.

## 2. Mollusca

The phylum Mollusca is made up of eight distinct classes and is highly diverse, though as of yet <1% of species have seen secondary metabolite investigation [52]—whilst testing of other compounds/molecules is similarly limited. Within this, proteins, lipids, and carbohydrates have seen particular research focus, with mussel lipids a well-established treatment for rheumatoid arthritis [53]. In terms of osteogenic bioactives, novel activity has been found—such as from abalone gastro-intestinal digests of *Haliotis discus hannai*. These partially digested extracts were investigated using an osteoblast-like cell line (MG-63), and subsequently were shown to increase alkaline phosphatase (ALP) and mineralisation levels [44]. RT-PCR and Western blot analysis found increased BMP-2 expression, thought to be a result of MAPK pathway activation. However, with the exception of this example, tests on nacre make up the majority of studies reporting osteogenic activity from molluscan derived material.

### Nacre

Nacre, often in its powdered form, features in a considerable body of research. Also known as mother of pearl, nacre is the lustrous aragonitic inner layer found on molluscan shells in taxa such as mussels and abalone. Like bone, nacre has both inorganic and organic components, with an organic shell matrix comprised of proteins, glycoproteins and polysaccharides which then serve as a template for calcium carbonate mineralisation [54]. It is this similarity that fuelled the idea that factors able to stimulate mineralisation and support healthy human bone may be contained within nacre.

Research on nacre has been conducted since the early 1990s, with initial in vitro work demonstrating its capacity to stimulate the mineralisation of human osteoblasts [55]. The most interesting of these early studies investigated the ability of nacre to aid bone reconstruction in human maxillary defects [56]. Here, nacre powder was mixed with the blood of patients and injected into the defect site of eight middle-aged female patients. The results showed no evidence of toxic effect and demonstrated enhanced mineralisation and good bio-dissolution of nacre within the area of injection. The significance of this discovery was not realised until a subsequent commentary was published [57], emphasising the remarkable ability of a raw and unrefined natural product to promote bone growth. Since this early work, there has been a surge of research effort, including in vitro and in vivo studies, as well as those specifically focusing on the proteins and mechanisms involved in enhancing cellular activity, making nacre an excellent case study of bioactive research.

A good example of the in vitro work conducted used water soluble matrix (WSM) extracted from the oyster *Pinctada fucata* [58]. This study demonstrated both the ability of nacre to enhance osteoblast differentiation (increased Col-I, osteocalcin, and ALP expression) and its ability to scavenge free radicals, suggesting an antioxidant potential that may also support bone regeneration. WSM has also been shown to increase bone mineral density (BMD) in an ovariectomized mouse model of osteoporosis [49], in part attributed to increased Runx2 and Fos-related antigen-1 expression as a result of JNK pathway stimulation in osteoblasts. Furthermore, the extract suppressed actin ring formation and RANKL-induced upregulation of c-Fos and NFATc1 in osteoclasts. Other in vivo work showed nacre implanted into rat femurs supported new bone formation, implant/bone fusion, and increased expression of numerous markers indicative of increased BMU action [59].

As the osteogenic potential of nacre is well established numerous studies have worked toward identifying active components within the extract. At a basic level, nacre WSM can be broken down into a number of fractions containing amino acids of varying size and composition [48]. Moreover, numerous proteins have been identified within the nacre of different species, many of which are thought to have roles in regulating bone tissue. For example, proteomic nacre analysis of the oyster *Crassostrea gigas* found four novel proteins thought to aid in shell mineralisation, with structures homologous to endogenous human proteins and with roles in osteogenesis [47]. Novel single proteins, such as p10 [60], P60 [51], and PFMG3 [61], have also been identified, all sourced from the pearl oyster *Pinctada fucata*. The three studies referenced above demonstrated the proteins’ ability to enhance crystallisation of calcium carbonate in vitro. An ability to enhance differentiation of osteoblast cell lines through various marker assays was also demonstrated, though the work of Wang et al. [61] was the most comprehensive. Additionally, nacre appears to contain proteinase inhibitors of varying molecular weights, which may help to conserve proteins important for processes such as mineralisation [62]. The matrix hosts low molecular weight molecules, which were shown to increase osteoblast mineralisation, Col-1 expression, and mRNA levels of Runx2 and osteopontin [63]. Overall then, these studies indicate that the effect of nacre, regardless of species, is due to the interaction of a range of bioactive molecules present within the extract. However, the exact structure and action of the majority remain to be identified.

## 3. Algae and Seaweed

One group of marine invertebrates which show promise as a source of bioactives is algae, particularly macroalgaes. Algae have a much greater bioactive diversity than nacre, including calcareous extracts, sulphated polysaccharides, and raw algal extracts.

### 3.1. Aquamin

Aquamin is a food supplement derived from the red algae *Lithothamnion corallioides*, and contains calcium, magnesium and 72 other trace minerals [30]. *L. corallioides* is fairly unique in that it is one of the few algal species to produce a calcareous skeleton. It is normally found on muddy or sandy substrates at less than 20 m in depth, in aggregations of unattached algae known as maerl beds [64]. It is from these beds, with a wide European (including western Irish and British shores) and more northern distribution, that maerl is collected before it is ground into the commercial product known as Aquamin. Currently, Aquamin is solely licenced to and produced by Marigot Limited (Marigot, Cork, Ireland).

Mineralising organisms like *L. corallioides* are an obvious choice when searching for extracts to promote bone health due to their structural similarities, and their tendency to contain similar key molecules [65] and high mineral content. The latter is the theoretical basis of Aquamin, as there are well established links between BMD and the intake of minerals such as calcium, magnesium, and zinc [66]. From a cellular perspective, O’Gorman et al. [67] investigated the ability of Aquamin to enhance mineralisation of a pre-osteoblastic cell line. Mineralisation did increase, though only at the latest time point (Day 28), whilst other markers of osteoblast activity, for instance proliferation, did not show significant differences between control and treatment. In vitro mineralisation is further enhanced when vitamin D is included with Aquamin treatment, as compared to levels in osteoblast cultures treated with only Aquamin or control solutions [68]. These studies appear to indicate that Aquamin has some potential to increase mineralisation, though further work is needed.

Aquamin was tested in vivo by inclusion in the food of mice given a Western style diet, which is known to detrimentally impact bone strength and mineralisation [69]. In this study, Aquamin negated diet-related bone defects, supporting its potential to maintain healthy functioning of the BMU. In another study, focusing on ovariectomized mice [70], Aquamin treatment increased mineral/matrix ratio and the hydroxyapatite content of trabecular bone when compared to a calcium carbonate treatment group. However, there was no significant difference in carbonate/phosphate ratio or hydroxyapatite levels of cortical bone, whilst those positive effects observed were fairly small—casting doubt on the eventual clinical potential of Aquamin. In vivo, the extract has also been shown to increase bone turnover in horses, as supplementation studies have shown enhanced levels of osteocalcin and type I collagen relative to limestone controls [71]. Interestingly, despite a lack of more basic studies, two randomised control trials have been conducted, aimed at assessing the efficacy of Aquamin supplementation in treating knee osteoarthritis [72,73]. These studies reported improvements in outcome measures of mean walking distance and range of motion for Aquamin treatment compared to controls. However, these results are limited due to the short study periods (12 weeks), lack of long-term follow up, and small sample sizes used. Similarly, a pilot trial investigated the impact of Aquamin F™ on markers of calcium metabolism, finding greater urinary clearance of calcium and a suppression of PTH concentration [74]. However, this study only looked at changes in these markers over a single 12 h period, meaning the long-term marker effects of Aquamin F™ treatment are yet to be established. Overall, further in vitro and in vivo studies are required before the true potential of Aquamin supplementation becomes apparent. Irrespective of its treatment prospects *L. corallioides* is unlikely to be a long-term resource, as it has a slow growth rate (1–2 mm/year) and is highly susceptible to sedimentation through natural or anthropogenic means [64].

### 3.2. Fucoidan

Within the algal group, fucoidan is one of the best studied extracts. Fucoidans are highly sulphated and fucose-rich polymers, found as a heavily branched and relatively high yield form in brown macroalgae and a more linear form in echinoderms [75]. These marine polymers are multifunctional, with a range of therapeutic uses, from anti-inflammatory to anti-viral [75].

In terms of its effects on bone tissue, it appears that the extract has both an anti-resorptive and osteogenic potential. Fucoidan extracted from the sea cucumber *Apostichopus japonicus* has been shown to inhibit osteoclastogenesis [43], whilst work by Kim et al. (2014) demonstrated the same effect with brown algal extracts added to bone marrow macrophage cultures. This reduction in osteoclast differentiation was attributed to inhibition of RANKL-dependent MAPKs and downregulation of c-Fos and NFATc1 transcription factors. However, the precise molecular mechanism through which this inhibition occurs is yet to be elucidated, which is a common limitation to studies on this polysaccharide. It is speculated that fucoidan binds to either RANKL or RANK to inhibit intracellular signalling, an action which may be facilitated by its sulphated regions [76].

With respect to osteogenic potential, low molecular weight fucoidan (LMWF) can promote osteoblast proliferation in vitro. This was measured as increases in the presence of osteoblast markers including ALP and type 1 collagen in 3D [77] and 2D culture [78]. The in vitro potential of fucoidan was also demonstrated in work by Pereira et al. [79], whereby extract treatment increased osteogenic differentiation as measured by ALP, osteopontin, Runx2, and calcium deposit formation in adipose tissue-derived stem cells. Related work confirmed increased osteoblast differentiation of mesenchymal stem cells upon fucoidan treatment, but also reported stimulation of angiogenic activities and associated pathways. Furthermore, greater blood vessel formation, along with a small promotion of bone formation, was also observed in a rabbit model of a calvarial bone defect [21]. Alternatively, a related study showed that fucoidan extracts are also capable of impairing angiogenesis in co-culture models relevant for bone vascularisation [80]. The two studies gave limited information on how each fucoidan extract was prepared, though both used a different brown macroalgae source species and fucoidan used by Wang et al. [80] is referred to as being ‘crude’. Differences in extract preparation may therefore explain their differing effects on angiogenesis.

One limitation of current published studies testing fucoidan is a lack of in vivo experimentation, which may simply be a function of the early developmental stage of much of this work. Of those few in vivo studies conducted to date, fucoidan appears to have a positive effect on bone formation. For example, LMWF was shown to increase femoral density and prevent microarchitectural deterioration in ovariectomized Sprague-Dawley rats [22], whilst bone density and bone ash weight of mice were also increased by LMWF treatment [81]. Further studies are required to fully detail the in vivo potential of fucoidan, though this initial work is promising. Alternatively, fucoidan could be applied to stimulate ex vivo osteogenic differentiation before in vivo re-implantation, though more testing is required to confirm this.

To date, the most comprehensive study completed on fucoidan is that of Kim et al. [82], which aimed to elucidate the complex mechanism by which the extract stimulates MSC differentiation into osteoblasts. ALP, mRNA levels of multiple other markers, and alizarin red-S (AR-S) staining was used to determine osteoblast differentiation and mineralisation, whilst Western blotting was also conducted, including antibodies for various intracellular signalling pathway proteins. The results indicated that BMP2-Smad 1/5/8 signalling was responsible for stimulating osteoblast differentiation through activation of ERK and JNK pathways.

### 3.3. Other Algal Bioactives

Whilst fucoidan is one of the better known bioactives, there are other marine algae extracts that also have an effect on bone homeostasis. Extracts from the brown algae *Sargassum horneri* are known to stimulate osteoblastogenesis and inhibit osteoclastogenesis in vitro in preosteoblastic and monocytic cell lines [24]. Similar in vitro and in vivo work was conducted using rat femoral tissues, which demonstrated the ability of *S. horneri* extracts to increase their bone calcium content [83] and inhibit bone resorption [84]. Such work complements in vivo tests, which indicate that *S. horneri* extracts have a preventative effect on bone loss in streptozotocin-diabetic rats [85]. Furthermore, there has even been a basic human trial investigating the effect of oral intake of the algae on bone metabolic markers [86]. Despite being limited by very small sample sizes, this study reported an inhibitory effect of the extract on bone resorption, as determined by decreased levels of circulating resorption markers such as TRAP, and is one of the few human trial studies conducted for any marine based compound. As with fucoidan, the main limitation to the *S. horneri* body of work lies in identifying the active component of the extract, which appears to be different for bone stimulation and suppression of resorption [83].

Another brown algae extract which has been tested both in vitro and in vivo is *Hizikia fusiforme* [27]. Specifically, a hot water by-product of the algae which contained high polysaccharide levels was shown to stimulate ALP activity and BMP-2 levels in mouse myoblast C2C12 cells. Furthermore, in vivo stimulations of skeletal activity were confirmed in zebrafish, ovariectomized mice, and mouse calvaria bones [27]. Similarly, a brown alga-based study investigated quinone derivatives from *Sargassum thunbergii*, finding that treatment with a crude extract containing these derivatives was able to enhance osteoblast differentiation [26]. Finally, fucoxanthin—a marine carotenoid present in brown seaweeds—has also been tested for osteogenic activity. Fucoxanthin administered to rats was shown to significantly decrease the oxidative stress index [23], but had no significant effect on ALP levels and only caused a limited reduction in alveolar bone resorption—despite significantly reducing RANKL levels. Similarly, a related study found that fucoxanthin had no effect on MC3T3-E1 (an osteoblast cell line) viability, but did stimulate apoptosis in osteoclasts [87]. As yet, research on this carotenoid is limited and so it is difficult to determine its in vitro effects, though it initially appears to inhibit osteoclast resorption rather than stimulating osteoblast activity.

In addition to Phaeophycae, green and red algae also contain examples of extracts which are able to stimulate osteogenic activity. Extracts from two marine green macroalgae, *Cladophora rupestris* and *Codium fragile*, were shown to increase osteogenic activity. These extracts were enriched in phenolic compounds and able to stimulate mineralogenic activity of a fish bone-derived cell line, as well as 1.5 fold increases in the operculum area of juvenile zebrafish [28]. Similarly, extracts from two red algae—*Plocamium cartilagineum* and *Ceramium secundatum*—increased activity of human bone marrow stromal cells (hBMSCs) and caused significant increases in the size of opercular bones in juvenile zebrafish [88]. Another red alga-derived osteogenic bioactive is floridoside, a glycerol glycoside metabolite of *Laurencia undulata* (amongst other red algae). Floridoside is known to promote differentiation of osteoblast D1 cells, as well as increasing ALP levels, mineralisation and expression of factors including type I collagen, Runx-2, and Osterix [29].

The studies discussed appear to indicate that algae may be a promising reserve of bioactive compounds, whilst the ease of sourcing these inshore and shallow water eukaryotes makes them a likely commercial source. However, it is apparent that more in vivo studies are needed, as well as a focus on the mechanisms involved and the active molecular component of algal extracts. One potential solution is the use of in vivo zebrafish models, as employed in two of the aforementioned studies [28,88], as these allow for inexpensive and rapid determination of osteogenic potential and the mechanisms/pathways involved in activity [89].

## 4. Assorted Taxa

**Figure 2:**
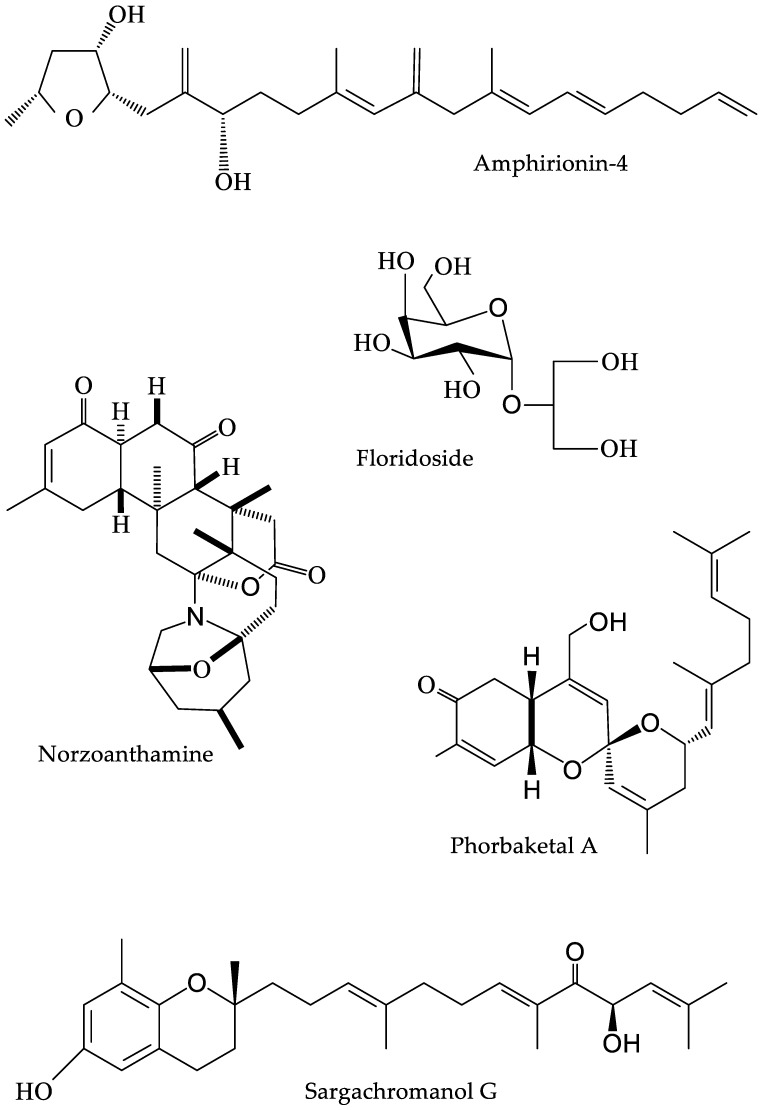
Chemical structure of major secondary metabolites included within this review.

There are numerous examples of taxa and extracts that have only undergone initial screening, many of which show osteogenic potential. The aforementioned abalone gastro-intestinal digests from *Haliotis discus hannai* promote osteoblast activity, but to date have only been reported in a single study [44]. Norzoanthamine (see Figure 2 for chemical structure), an alkaloid isolated from a colonial zoanthid, *Zoanthus* sp., is also known to have anabolic effects on bone, accelerating the formation of a collagen-hydroxyapatite composite [38]. It is particularly effective at binding collagen and has been previously shown to suppress decreases in various parameters indicative of bone health in ovariectomized mice [90]. Another natural marine compound with osteogenic potential is Phorbaketal A, derived from the marine sponge *Phorbas* sp. This bioactive was shown to stimulate osteoblast differentiation in mesenchymal stem cells, predominantly through activation of the extracellular signal-regulated kinase (ERK) pathway [37]. Mussel adhesive proteins are also of interest as they are known to increase cell proliferation and osteogenic differentiation, and can be used to easily coat graft materials [46]. This, along with their biocompatibility and biodegradability, makes them promising adjuncts to synthetic grafts. These preliminary studies (and those of Section 4.1 and Section 4.2), looking at various markers of cell-stimulation potential, highlight the diversity of marine bioactives and indicate that it is primarily research effort which determines success within this field.

### 4.1. Microalgae

There are a number of bioactives that have been isolated from microalgae that show osteogenic potential. The polyketide amphirionin-4 was isolated from *Amphidinium* sp.—a marine dinoflagellate. Application of only 0.1 ng/mL amphirionin-4 stimulated a 950% promotion in murine bone marrow stromal ST-2 cell proliferation [33]. Similar proliferative effects were also observed from experiments using amphirionin-5, with a 282% proliferation increase in murine bone-marrow derived stromal ST-2 cells and a 320% increase for MC3T3-E1 cells [91]. Another marine dinoflagellate derived compound is symbioimine, from *Symbiodinium* sp. This secondary metabolite is anti-resorptive in nature, inhibiting the differentiation of RAW264 cells into osteoclasts [32]. Biselyngbyaside, a macrolide compound from marine cyanobacteria *Lyngbya* sp., also inhibits osteoclastogenesis and induces apoptosis in mature osteoclasts [31]. Similar results are presented for a range of extracts, such as microalgae byproducts [34] and a depsipeptide (largazole) sourced from cyanobacteria [92]. Another interesting prokaryote is *Alteromonas infernus*, which is a deep sea species known to produce a high molecular weight polysaccharide. This oversulphated polysaccharide increased chondrogenesis in vitro, via the MAPK pathway, and may consequently be a potential therapy for cartilage repair [35].

### 4.2. Corals

Corals, of the phylum Cnidaria and class Anthozoa, are another group which have received surprisingly little attention. This phylum includes mineralising species, which have a skeleton comprised of calcium carbonate either in the form of calcite, aragonite or a mixture of the two, as with the Scleractinian or stony corals [93]. Like those taxa containing nacre, coral skeletons also include an organic phase, comprised of proteins, polysaccharides, lipids, and glycosaminoglycans [65]. Marine invertebrates such as corals have a long evolutionary history of developing proteins and genes that govern biomineralisation, many of which are highly conserved and analogous to human variants [65]. In scleractinians, both soluble and insoluble components of the organic matrix can influence calcium carbonate precipitation [94]. More specifically, multiple species express BMP2/4 orthologs, which show specificity to murine BMP receptors despite the taxonomical distance between the two groups [95]. This indicates incredible conservation of the protein sequence over time and suggests other proteins of skeletal importance may also be present within coral tissue. The presence of these bioactives is also supported by the regenerative ability of corals, many of which show relatively rapid skeletal repair, particularly those species living in shallow water, high-energy environments [96]. Despite their significance as a potential source of bioactive molecules, research using coral extracts is limited. Instead, the majority of research has focused on their potential as a scaffold material for graft procedures, due to their structural and compositional similarities to human bone [14].

## 5. Challenges to Using Marine Bioactives

Conservation concerns are an important consideration when looking for potential bioactive sources. Taking corals as an example, it is well known that the world’s populations are a serious conservation issue [97], with both cold and warm water species showing declining abundance and many listed in the Convention on International Trade in Endangered Species (CITES) list [98]. Reasons for the decline are varied, including increasing water temperature and ocean acidification associated with climate change, as well as damage from trawling, dynamite fishing, aquarium stocking, and various forms of anthropogenic pollution [97]. This, coupled with slow growth rates, a long time to maturity, and relatively limited ecological niches, means further declines in coral numbers are likely. The sustainability of these invertebrates as a source of bioactives is therefore questionable, although some species such as *Millepora dichotoma* are more abundant and show promise for aquaculture production [39]. Though corals are perhaps an extreme example in terms of sustainability, many marine invertebrates suffer from low abundance or long development cycles. In these cases bacterial cultures (modified to express the bioactive) or synthetic production are potential solutions, though complex molecule structures often make this challenging [99].

Aquaculture may become a necessity if commercial bioactives are to be sourced from marine organisms, as yields of target compounds are often very low, needing a large quantity of raw material for significant extraction [99]. In particular, secondary metabolite yields tend to be very low, though structural and non-metabolic proteins (like many of those with skeletal importance) tend to have higher yields [65]. Even if yield is not an issue, aquaculture comes with a whole host of other challenges involved in maintaining a large-scale culture system. Algal aquaculture requires consideration of temperature, light levels, hydrodynamic and general stress levels, as well as many other factors [100]. These, coupled with harvesting and processing complexities, mean that aquaculture systems are often inefficient and not cost-effective. Another concern is the spread of invasive species, as aquaculture is thought to be a major source of introduction for invaders—such as the brown alga *Undaria pinnatifida* [101]. Finally, it is also necessary to consider the costs and difficulties associated with collecting enough quality specimens in the first place, an issue which is further complicated when sourcing deep sea species. Advances in technology, such as the development of sophisticated remotely operated vehicles (ROVs), is beginning to make collection of these specimens more feasible. However, production and use of ROVs is expensive and often involves long voyages and use of a large vessel—further raising costs. In shallow water areas (<50 m depth), SCUBA diving is still the most effective method of sample collection. However, despite being more affordable, diving for samples is time-consuming, particularly compared to terrestrial sampling methods.

## 6. Conclusions

Osteoporosis is particularly prevalent in aged populations, a fact which is daunting considering estimations that by 2030 20% of Europeans and 30% of the US population will be over the age of 65 [102]. It is therefore in the best interests of patients, governments and health services to develop effective treatment options now. Furthermore, should an effective osteogenic bioactive be found, there are many more clinical applications for such a therapy. This review has demonstrated that marine organisms, such as molluscs and algae, are a highly promising source of osteogenic compounds. Algal sources in particular show excellent potential, as there are already indications of activity with limited study effort. This, coupled with other factors like ease of sampling, their great diversity, large secondary metabolite production, and quick growth times, make them excellent candidates as target source organisms.

Despite the possibility for drug discovery within this field, current research is highly limited. Compared to the huge diversity of marine organisms, only a fraction have been tested for their bioactivity, and even fewer specifically for osteogenic activity. More research is required across all taxonomic groups. At present, in vitro descriptions of activity are generally well detailed, though many studies lack identification of the bioactive structure and mechanism of activity. Furthermore, in vivo work is limited and needs greater focus in future studies, whilst human trials are even rarer—though arguably this is a reflection of the current phase of much research within the field.

In conclusion, marine bioactives are a highly promising reserve of osteogenic compounds, as well as bioactives in general. The dearth of research in this area increases discovery potential, making it an exciting field to conduct research in. Greater study effort would likely lead to the discovery of effective treatment options for osteoporosis and other conditions, reducing health care burden and improving patient quality of life.

## Figures and Tables

**Table 1:** Summary table showing the genus, species, general description, and extract type of key invertebrates that have been tested for their osteogenic effect in cell culture or relevant in vivo models. This demonstrates the range of taxa investigated within this field, which are also referred to in the text.

Genus and Species	General Description	Extract/Bioactive	Reference Example
Numerous assorted species	Brown algae	Fucoidan	[21]
Numerous assorted species	Brown algae	Fucoidan (low molecular weight)	[22]
Numerous assorted species	Brown seaweed	Fucoxanthin	[23]
*Sargassum horneri*	Brown algae	Raw extract	[24]
*Sargassum siliquastrum*	Brown algae	Sargachromanol G	[25]
*Sargassum thunbergii*	Brown algae	Quinone derivatives	[26]
*Hizikia fusiforme*	Brown algae	Water by-product	[27]
*Cladophora rupestris*	Green algae	Crude extract	[28]
*Codium fragile*	Green algae	Crude extract	[28]
*Laurencia undulata*	Red algae	Floridoside	[29]
*Lithothamnion corallioides*	Calcareous red algae	Aquamin	[30]
*Lyngbya* sp.	Cyanobacteria	Macrolide	[31]
*Symbiodinium* sp.	Dinoflagellate	Symbioimine	[32]
*Amphidinium* sp.	Dinoflagellate	Polyketide	[33]
*Nannochloropsis oculata*	Microalgae	Peptide	[34]
*Alteromonas infernus*	Prokaryote	Polysaccharide	[35]
*Symploca* sp.	Cyanobacterium	Largazole (depsipeptide)	[36]
*Phorbas* sp.	Sponge	Phorbaketal A	[37]
*Zoanthus* sp.	Zoanthid	Norzoanthamine	[38]
*Millepora dichotoma*	Hydrocoral	Bioactive material	[39]
*Porites lutea*	Stony coral	Bioactive material	[40]
*Porites lutea*	Stony coral	Biomatrix	[40]
*Synularia polydactyla*	Alcyonarian coral	Proteins	[41]
*Xenia elongate*	Soft coral	Coral cells	[42]
*Montipora digitata*	Hard coral	Coral cells	[42]
*Apostichopus japonicus*	Sea cucumber	Fucan sulphate	[43]
*Haliotis discus hannai*	Abalone	Digested intestines	[44]
*Haliotis laevigata*	Abalone	Perlucin protein	[45]
*Numerous assorted species*	Mussels	Adhesive protein	[46]
*Crassostrea gigas*	Oyster	Protein Nacre (water soluble matrix)	[47]
*Pinctada maxima*	Pearl oyster	Individual proteins Low molecular weight molecules.	[48]
*Pteria martensii*	Pearl oyster	Nacre (water soluble matrix)	[49]
*Pinctada margaritifera*	Oyster	Proteinase inhibitor Proteins Nacre (water soluble matrix)	[50]
*Pinctada fucata*	Akoya pearl oyster	*Pinctada fucata* mantle gene 3 Protein p10 and other novel proteins	[51]
